# Recent Progress in Applicability of Exercise Immunology and Inflammation Research to Sports Nutrition

**DOI:** 10.3390/nu13124299

**Published:** 2021-11-28

**Authors:** Katsuhiko Suzuki

**Affiliations:** Faculty of Sport Sciences, Waseda University, 2-579-15 Mikajima, Tokorozawa 359-1192, Japan; katsu.suzu@waseda.jp; Tel.: +81-4-2947-6898

**Keywords:** exercise, training, immunity, inflammation, metabolism, oxidative stress, muscle damage, antioxidant, anti-inflammatory substances, functional foods

## Abstract

This article focuses on how nutrition may help prevent and/or assist with recovery from the harmful effects of strenuous acute exercise and physical training (decreased immunity, organ injury, inflammation, oxidative stress, and fatigue), with a focus on nutritional supplements. First, the effects of ketogenic diets on metabolism and inflammation are considered. Second, the effects of various supplements on immune function are discussed, including antioxidant defense modulators (vitamin C, sulforaphane, taheebo), and inflammation reducers (colostrum and hyperimmunized milk). Third, how 3-hydroxy-3-methyl butyrate monohydrate (HMB) may offset muscle damage is reviewed. Fourth and finally, the relationship between exercise, nutrition and COVID-19 infection is briefly mentioned. While additional verification of the safety and efficacy of these supplements is still necessary, current evidence suggests that these supplements have potential applications for health promotion and disease prevention among athletes and more diverse populations.

## 1. Introduction

**Figure 1:**
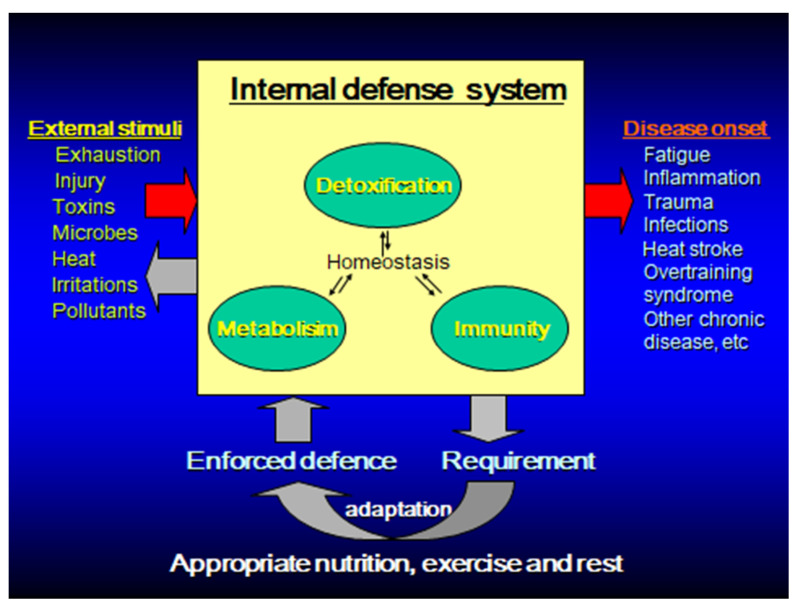
Homeostasis enforcement model for disease prevention through functional foods/supplements, exercise and rest by enhancing body’s innate defense systems.

Immunity is a biological defense mechanism that attempts to maintain homeostasis by eliminating foreign bodies, such as microorganisms, abnormal substances, waste products, and diseased cells in the body. In immunocompromised states, such as malnutrition and old age, infections and malignant diseases are more likely to occur. On the other hand, excessive immune response (inflammation) may cause autoimmune diseases and allergic diseases, and may destroy healthy normal tissues [1,2]. Thus, the immune responses have both merits and demerits. The immune system must be appropriately maintained and managed to respond to foreign bodies without deficiency or excess and avoid infections and inflammatory diseases [1,3,4]. In the context of exercise and sport, exhaustive physical activity (either acute or chronic) can perturb the immune system, causing an imbalance and prompting the immune system to either over- or under-perform. Conversely, if immunity and inflammation can be controlled, various diseases can be prevented and improved, leading to the maintenance and promotion of health (Figure 1).

Although the relationship between nutrition and immunity has been studied for a long time, the relationship between exercise and immunity has not been fully elucidated until the last few decades. The International Society of Exercise and Immunology (ISEI) (https://exerciseimmunology.com/, accessed on 26 November 2021), a representative academic organization, was established in 1993. Thus, exercise immunology is a relatively new field of study developing with the involvement of related areas, such as inflammation, ageing, and nutrition. Exercise immunology is deeply related to the issues of today, such as countermeasures against problems with the ageing population, and an increase in chronic diseases, as well as in the diagnosis and prevention of various diseases (such as metabolic syndrome and COVID-19) and conditioning of athletes [1,3,4,5,6,7,8,9,10]. Thus, it can be applied to both sport and broader society.

This article shares findings from our experimental research on exercise immunology as it relates to inflammation and nutrition. It is not intended to be comprehensive nor exhaustive; rather, it is intended to be a representative sampling of current research in the field. Topics are considered with athletic applications, including: how ketogenic diets (KD) might alter both immune function and metabolism [11,12] (Section 2); how vitamin C, sulforaphane (SFN) [13], and taheebo polyphenol might bolster antioxidant defense (Section 3); how colostrum and a concentrated form of hyperimmunized milk termed “immune proteins” (IMP) [14] might prevent systemic inflammation or organ damage (Section 4); how 3-hydroxy-3-methyl butyrate monohydrate (HMB), a leucine derivative [15,16], may influence muscle strengthening in athletes (Section 5); and the importance of both diet (including fasting condition) and exercise in athletes following pandemic protocols, such as the recent COVID-19 lockdown [8] (Section 6). Finally, some overarching conclusions and potential future directions are suggested. 

## 2. Role of Ketogenic Diet and Carbohydrate Intake to Control Inflammation and Transform Energy

Excessive intake of carbohydrates and fats is a hindrance in competitive sports, requiring weight control due to the risk of lifestyle-related diseases, such as obesity [3,4,17,18,19]. In addition, the amount and timing of carbohydrate intake should be carefully considered to avoid hypoglycemic symptoms (insulin shock) during exercise due to the increased secretion of insulin [20,21]. Furthermore, it is necessary to take into account the individual’s constitution, the amount of physical activity, rest, and timing of energy intake with meals [20,21,22]. While glucose oxidation requires 11 chemical reactions to produce energy, ketone bodies, metabolites of fat, can produce adenosine triphosphate (ATP) quickly with only three chemical reactions. Moreover, the energy density and possible storage amount of fat are promising. A KD can also make fatty acid mobilization and utilization more easily. In addition, lipid oxidation produces less reactive oxygen species (ROS) during the metabolic process than glucose oxidation. Thus, KD may improve endurance through metabolic transformation, and at the same time, it may prevent organ injury caused by ROS production during exercise and accelerate recovery from fatigue [11,23,24].

KD utilize fat and lipid metabolism as the main energy source while limiting carbohydrate intake [11,23,24]. This diet has several advantages and disadvantages. One benefit is that limiting carbohydrate consumption can help regulate levels of serum triglycerides, blood glucose, and insulin [25]. Such benefits may be more critical for individuals with insulin resistance, for whom ingesting large amounts of carbohydrates can be hazardous, as was shown in a 14-month research study in obese diabetic adults [26]. Other benefits include: reducing fasting blood glucose levels, fat oxidation, inflammation, and oxidative stress resulting from exercise; decreasing cardiovascular and cardiometabolic risk factors; preserving glycogen reserves; and, maintaining and even losing weight in those with high blood fats [27]. Also, this diet is favored by athletes who need to lose weight and increase performance [28]. Regardless of protein, fat, or calorie intake, it is thought that participants should consume less than 20 g of carbohydrates per day to reap these advantages [29], but athletes usually achieve the desired results by consuming less than 50 g [30].

Almost all studies on KD have been short-term (up to two years), with just a few studies reporting long-term consequences of this type of diet. Common adverse effects seen in short-term studies include dizziness, vomiting, difficulties with exercise tolerance, nausea, constipation, headache, sleeplessness, and exhaustion. Of course, remission from these symptoms can take anywhere from a few days to a few weeks, and it is usually advised to drink enough electrolytes and fluids to counteract the diet’s unwanted effects [31]. Long-term intake of KD increases the production of ketone bodies in the liver and other organs, and skeletal muscles and the brain adapt to use ketone bodies as an energy source (keto-adaptation) [11]. Though the number of research studies is limited, adverse effects seen in long-term studies (greater than two years) include kidney stones, hypoproteinemia, vitamin and mineral shortages, and hepatic steatosis. KD can also induce side effects such as anemia and liver dysfunction due to fatty liver, which should be monitored by regular blood tests [11,31]. 

In addition, it was recently reported that KD, either alone or combined with training, had no beneficial effects in the intensive exercise-evaluation model, suggesting that KD may be promising in terms of improving endurance in low-to-moderate-intensity exercise, but may not be an optimal choice for those undertaking in high-intensity exercise [32]. Anaerobic or high-intensity exercise is a type of intense short-term activity that lasts less than 2 min. During short-term activity, immediate energy supply is needed, while body synthesizes ATP/energy very quickly using a phosphagen system and provides maximal power for an acute burst of activity [33]. Because the lactic acid and phosphagen systems use it for energy, skeletal muscle glycogen is essential in this action. On the other hand, intense activity is frequently accompanied by many muscular contractions, resulting in muscle damage. As a result, a steady supply of glycogen to the muscles supplied by dietary carbohydrates can play an essential role in the regeneration and repair of these muscle fibers [34]. A sufficient supply of necessary amino acids, in addition to supplying appropriate muscle glycogen, aids in the better and quicker healing of injured muscle fibers. Given this, KD which provide adequate protein at around 15% of daily calories can constitute a good alternative to preventing amino acid deficits in skeletal muscle fibers. The low carbohydrate content of this diet, on the other hand, causes muscle glycogen regeneration to be hampered. As a result, during high-intensity activity, the KD is rarely employed [35].

**Figure 2:**
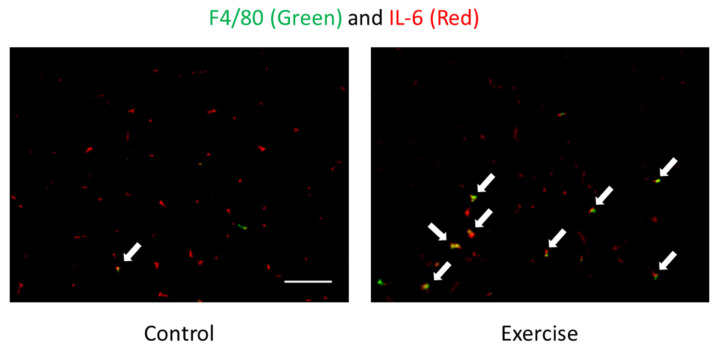
Localization of F4/80 (monocytes/macrophages) (green) and IL-6 (red) of skeletal muscle after exercise detected by immunofluorescence staining [7,40]. Arrows (yellow) indicate F4/80 and IL-6 double positive cells. The signals of IL-6 were mainly observed in the interstitial space. Exercise increased F4/80 and IL-6 double positive cells but not IL-6 positive myocytes, suggesting that IL-6 is produced by immune cells. Scale bar is 100 µm.

During endurance exercise, skeletal muscle produces interleukin-6 (IL-6), which is called a myokine because it is produced by muscle and involved in energy metabolism, such as glucose uptake and lipolysis [36]. We have also reported that IL-6 may be associated with endurance exercise performance [37], lipolysis [38], and metabolic changes in keto-adaptation [39]. However, immunohistochemical staining results indicate that IL-6 production during endurance exercise mainly occurs in monocytes/macrophages in the interstitial space rather than in myofibres themselves (Figure 2). This suggests that IL-6 is not a myokine but a classical monokine (monocyte-derived cytokine), whereas immune cells may be involved in energy metabolism during endurance exercise [40]. However, carbohydrate intake does not affect the infiltration of IL-6-producing monocytes/macrophages into skeletal muscle by exercise, which is different from the previous findings that carbohydrate intake suppresses IL-6 production during exercise; therefore, there is still room for further study [7,22,40].

In addition, carbohydrate intake after endurance exercise does not adversely affect the inflammatory response and contributes to glycogen recovery [20]. Jürimäe and colleagues used female rowers as volunteers in their study, who were asked to row incrementally until fatigue on a wind resistance-braked rowing ergometer for an hour. This endurance activity increased blood IL-6 [41]. In research, the three athletes competed in ironman and half-ironman events, and their inflammatory markers, particularly IL-6, increased towards the end of the race [42,43]. Well-trained triathletes competed in an ironman triathlon competition (3.8 km swimming, 180 km cycling, and 42.2 km running) in a study comparable to the previous one [43,44]. In addition, Robson-Ansley and colleagues recruited male athletes to complete a six-day cycling challenge (468 km), and a rise in IL-6 was also detected immediately after exercise on the first day of this research [45]. In another study, healthy volunteers’ inflammatory markers (IL-6) did not alter after 16 weeks of endurance training [46]. In addition, Sponder and colleagues found a reduction in IL-6 following long-term endurance training in their study [47]. It is true that circulating IL-6 levels increased by more than 100 times following full marathon races [2,36,37,48], but decreased in longer endurance exercises, such as ironman triathlon races [42,43,44], and even downregulated by long-term training [47].

To summarize, a KD may exert its positive influence, including enhancing exercise capacity, and alleviating exercise-induced inflammation and oxidative stress, whereas it is employed to suitable situations, for instance, low-to-moderate-intensity sports. Furthermore, consideration of using substitution of KD, such as exogenous ketone bodies to achieve ketosis, may further contribute to the field of athletes’ wellness, and it is necessary to examine the effectiveness and safety precisely in the future research.

## 3. Enhancement of Endogenous Antioxidant Defense Mechanisms against Exercise-Induced Oxidative Stress (OS)

When the quantity of reducing compounds in a cell is substantially lowerr than the number of oxidized compounds, a redox reaction becomes unbalanced, resulting in the formation of ROS and OS in the body’s biological functions [49,50,51]. When ROS are produced in modest amounts, cellular adaptation and even some cellular redox balance are both promoted [49,50,51,52,53]. ROS contribute to the maintenance of proper muscle contractions by interacting with the troponin protein complex and calcium (Ca^2+^) secretion sites in the sarcoplasmic reticulum (SR) [52,53,54]. ROS overproduction might overload the muscle’s ability to produce strength and contraction under stressful conditions, such as participating in physical activities with variable intensities of anaerobic and resistance training [55]. It would also have a detrimental effect on athletic performance [56]. As previously mentioned, the absence of reducing compounds as a result of ROS production is a cellular destructor. Exogenous (such as taking antioxidant supplements including vitamin C) and endogenous (such as the antioxidant enzymes catalase (CAT), superoxide dismutase (SOD), and glutathione peroxidase (GPX)) antioxidants are present in the body’s cells [55,56,57]. Antioxidants are essential for preventing ROS-induced damage [55,56,57,58]. As a consequence, they typically transform these reactive species into less reactive ones [55,56,57,58,59].

The production of ROS during exercise, on the other hand, induces skeletal, muscular damage, which eventually appears as delayed-onset muscular soreness (DOMS) as one of the damage’s side symptoms [60,61,62,63,64,65]. Morrison and colleagues employed healthy young men as their research respondents in one study. For 28 days, they were administered antioxidant supplements of vitamins C and E (1 g and 400 IU per day, respectively). They underwent acute aerobic exercise and also had their skeletal muscle changes evaluated. According to the research, antioxidant supplements could not diminish the OS produced in skeletal muscle by performing acute aerobic exercise [66]. He and colleagues supplied vitamins C and E (1000 mg and 400 IU per day, respectively) to trained men for 14 days. They conducted a 40-min downhill run and concluded that these vitamins alleviated DOMS [67]. Gabrial and colleagues employed active men who exercised recreationally as subjects in another experiment. These people were put to a lot of inconsistent exercises (2 to 3 times a week). For three months, they were also given 500 mg of vitamin C each day. Vitamin C decreased exercise-induced muscle damage and oxidative stress, according to the study [68]. Evans and colleagues investigated untrained individuals’ muscle force and athletic performance in a research study. The subjects were given 500 mg of vitamin C and resistance training for 28 days. The researchers concluded that reducing OS from exercise enhanced muscular force and athletic performance [69]. Jalalvand and colleagues selected eccentric contraction in two hands as the exercise test in another experiment. Healthy people consumed 750 mg of vitamin C per day for four days in this study. Ultimately, they found that taking the supplement diminished exercise-induced muscle damage [70]. Vitamin C supplements (100 and 200 mg per day) and placebo were administered to healthy non-athlete women before exercise and 24 and 48 h after training in an investigation. These subjects were exposed to eccentric contractions. Finally, it was revealed that the DOMS induced both by the supplement and placebo groups were the same [71]. 

One of the determinants of endurance performance during strenuous exercise is aerobic metabolism capacity, which particularly impairs skeletal muscle contractile function and inevitably leads to muscular exhaustion. Muscle weariness then results in free radicals and metabolic disturbances, such as lactic acidosis [72]. Nutrient supplementation is one option to enhance an athlete’s endurance performance. Decreased serum triglycerides, blood pressure, blood glucose and insulin levels, and visceral adipose tissue are all associated with high endurance performance. Polyphenols with antioxidant properties are crucial among these nutrients for boosting endurance capacity by enhancing mitochondrial biogenesis and fatty acid intake and reducing oxidative stress [73]. Anti-inflammatory properties are another feature of polyphenols. Natural polyphenols have been built to demonstrate up to approximately 30% of anti-inflammatory pharmaceuticals developed in the 1980s. Inflammation caused by bacteria and other pathogens infecting the human body, in which case the immune system fights the infections [74]. Obesity, which increases triglyceride production in adipose tissue and leads to an excess release of free fatty acids, is one of the most essential factors in producing local inflammation in the body. Cyclooxygenase (COX)-2 catalyzes the transformation of arachidonic acid to thromboxane and prostaglandin (PG) E_2_ (PGE_2_) in response to inflammation. COX-1, on the other hand, produces PGs, which are engaged in a variety of hemostatic tasks, such as renal blood flow control and platelet function integrity. The gastrointestinal system is harmed when COX-1 is absent [75]. By eliminating free radicals, such as superoxide anions, polyphenols also aid in maintaining the body’s immune system and the prevention of cancer cell development. On the other hand, these nutrients contribute to the control of blood glucose transporter gene expression and the lowering of blood glucose levels [76]. However, some polyphenols are effective for skeletal muscle antioxidation but have side effects, such as hepatic dysfunction [73,76,77]. As a result, we must be cautious regarding the type, dosage, and timing of antioxidant ingestion. 

The aqueous extract of taheebo (derived from the inner bark of Tabebuia avellanedae) is suggested to have anti-inflammatory, anti-fatigue, anti-obesity, and anti-cancer properties. Taheebo also aids in the immune system’s upkeep. This plant has mostly been found in Central and South America [73,76]. The influence of taheebo polyphenol on endurance capacity was investigated for the first time in our study [73]. All mice used in the study were C57BL/6J mice. The mice were provided taheebo polyphenol extract and were given endurance exercise. Yada and colleagues determined that consuming this polyphenol eliminates free radicals, which regulates skeletal muscle glycogen levels and speeds up the gluconeogenesis process, resulting in improved mouse endurance capacity [73]. 

Sulforaphane (SFN) is another antioxidant supplement that is abundantly contained in broccoli sprouts and fights against OS via nuclear factor E2 factor-related factor (Nrf2) [13,78]. It also has immunomodulatory, anti-inflammatory, antibacterial, anti-carcinogenic, cardioprotective, and neuroprotective effects [13,78,79,80]. Inflammation is induced by exogenous and endogenous stimuli. Furthermore, nuclear factor-kappa B (NF-κB) is a transcription factor that regulates various genes responsible for inflammatory responses, and SFN inactivates NF-κB and contributes to anti-inflammatory effects. In our animal study, it was demonstrated that pre-administration of SFN prevented the production of inflammatory cytokines and hepatic dysfunction induced during strenuous exercise, due to the induction of antioxidant enzymes via Nrf2 [78,79,80]. Since no specific side effects have been reported in human studies, it is expected that the various effects and safety of SFN will be further investigated in the future [13,78,79,80]. We also looked at organ damage mediated by intensive exercise in mice in one trial. Two hours before exhaustive exercise, mice were given a 50 mg/kg body weight SFN. Finally, we arrived at the conclusion that SFN would treat inflammation spurred on by rigorous activity. The important reason for this improvement was the activation of the Nrf2/HO-1 signal transduction pathway as a result of antioxidative defense responses [79].

## 4. Prevention of Exercise-Induced Intestinal Injury and Systemic Inflammation by Colostrum and IMP

Intense exercise causes inflammation, immunosuppression, gastrointestinal disorders, and other health problems [7,81,82], and colostrum has been used to prevent the increased intestinal permeability that causes these problems [83,84]. Colostrum contains high-quality proteins necessary for growth and antibodies necessary for infection protection, and it has also been proven to inhibit the production of pro-inflammatory cytokines [85,86]. Bovine colostrum is the first milk produced after birth, and it contains peptides with antimicrobial activity, nutrients, growth factors, and immunoglobulins [86]. Pregnant women in their seventeenth week were enrolled as subjects by Aparicio and colleagues until delivery. The goal of this research was to see how exercising during pregnancy affected the inflammatory markers in colostrum. This research used resistance and aerobic training program (3 sessions of 60 min per week). During colostrum, the results showed that this activity reduced pro-inflammatory and anti-inflammatory profiles (just like TNF-α, IL-1β, IL-6, IL-8, and IL-10) [87]. Immune system markers, such as TNF-α and IL-1, IL-2, IL-10, and IL-13, were measured in elite basketball players in a recent study by Skarpanska-Stejnborn. For six months, these participants were additionally given 6.4 g of bovine colostrum every day. They undertook a rigorous physical activity regimen before supplementation, as well as three months and six months after supplementation. The findings of this investigation revealed that using bovine colostrum did not change the dynamics of immune function markers [88].

Furthermore, hyperimmunized milk, obtained from cows vaccinated against specific pathogens (26 antigens including *E. coli*, *Salmonella*, and *Staphylococcus aureus*), contains large amounts of antibodies against pathogens and anti-inflammatory effects to protect intestinal functions, and IMP is a concentrated form of hyperimmunized milk. We investigated organ injury and inflammatory response in male runners to determine whether IMP has a protective effect against exercise-induced inflammation and organ injury [14]. The results suggested that urine-specific gravity and urine osmolality decreased, and urine concentrating ability in the kidney dropped after running an all-out time trial of 3000 m [89]. Still, urine-concentrating power was maintained in the IMP-treated group, making them less prone to dehydration [14]. In addition, we proved that eight weeks of IMP administration suppressed the elevation of intestinal fatty acid-binding protein (I-FABP), which is a marker of intestinal injury and inflammatory cytokines after all-out running, and that IMP intake can prevent exercise-induced intestinal damage and inflammation [7,14,90].

## 5. Exercise-Induced Muscle Damage, Strengthening and HMB

We have reported for more than 20 years that neutrophils and macrophages are involved in muscle damage, inflammation, and oxidative stress caused by intensive exercise [7,91,92,93,94,95,96,97]. In one of our studies, we reported that the secretion of inflammatory cytokines and the activation of various neutrophil activation markers, such as lactoferrin (LTF) and myeloperoxidase (MPO), increased after strenuous exercise, especially marathon running [95]. On the other hand, it has been shown that ROS production in neutrophils can be modulated through regular and single bouts of exercise [92,93]. Furthermore, there are defense mechanisms against OS and inflammation in the body [94,95], and it has been reported that not only endocrine factors (such as adrenaline and cortisol), which have anti-inflammatory effects, but also anti-inflammatory cytokines (such as IL-1 receptor antagonist and IL-10) are induced during exercise [2,7,37,65]. In addition, we have found that such antioxidant and anti-inflammatory responses can be partially induced by functional foods such as curcumin [97,98,99,100], and we believe that measures targeting inflammation control are important to prevent muscle function deterioration and fatigue caused by intense exercise, and we are working on the selection of candidate substances and analysis of the mechanisms such as Nrf2 [7,13,78,79,80].

HMB, a derivative of leucine contained in branched-chain amino acids (BCAA), activates protein synthesis in skeletal muscle and also inhibits oxidative stress and inflammation [9,15,16,101,102]. HMB has a positive impact on the body by decreasing the effectiveness of intracellular proteolytic pathways and enhancing the membrane integrity of myocytes, such as the sarcolemma. HMB forms a partnership with a protein called ubiquitin because of its antagonistic effect on protein breakdown processes [101,102,103]. Activation of the mTOR kinase pathway by HMB, on the other hand, can contribute to muscle protein anabolism by boosting the transcription level of the insulin-like growth factor-1 (IGF-1) gene [101]. The mTOR kinase pathway is important because it aids cell proliferation, transcription, growth, and translation in muscle protein production [101,102,103].

In particular, HMB-free acid (HMB-FA) has excellent intestinal absorption and is expected to have immediate effects, but a double-blind, randomized, controlled trial using 3 g of HMB-FA and placebo per day for 6 weeks after resistance training showed that HMB-FA increased muscle strength and the secretory response of growth hormone and IGF-1, which have anabolic effects on protein [15,16,102]. In addition, plyometric training, which has been attracting attention as a method of increasing instantaneous power, such as jumping power [101,103,104], causes muscle damage and inflammatory oxidative stress when the load is high, but HMB-FA intake was shown to prevent these problems [16,101,102,103]. Thus, HMB may have led to the enhancement and improvement of muscle function, not only by promoting muscle protein synthesis but also by preventing muscle damage through its antioxidant and anti-inflammatory effects. As an application of the above findings, HMB is expected to be effective in the prevention of age-related muscle weakness (sarcopenia) and arteriosclerotic diseases, and this will require further the accumulation of knowledge in the future, such as combining it with various types of exercise [9,105]. Furthermore, our studies report that decreased muscle mass and strength could occur due to systemic age-related inflammation in skeletal muscle, suggesting that this is due to ROS accumulation and an increase in inflammatory mediators [106].

One of the questions that has recently arisen for researchers in exercise and health fields is whether excessive injection of peptide hormones, combined with resistance training, can weaken the immune system. This is a question for which there is no conclusive solution. However, several studies have examined this issue. One study found that free radicals and OS may accumulate in bodybuilders who do strenuous resistance exercises. An increase in OS in the body provides the basis for increasing circulating concentrations of elastase, MPO, and neutrophils, ultimately increasing inflammation in the body and accelerating the weakening of the immune system [97,107]. In this regard, Mohammadjafari and colleagues used male bodybuilders as subjects in their research. These subjects were injected with growth hormone and IGF-1 for one year. They underwent strenuous resistance exercises (five sets with 80% of one-repetition maximum). They eventually concluded that OS markers, such as 8-hydroxy-2-deoxyguanosine (8-OHdG), malondialdehyde (MDA), and nitric oxide (NO), increased in these bodybuilders, which were associated with inflammation [107].

## 6. Exercise and Nutrition under the Spread of COVID-19 Infection and Consequent Lockdown Restrictions

At the end of October 2021, the worldwide number of infected people was about 243 million and the number of deaths was about 4.9 million, and those data continued to increase (https://www.who.int/publications/m/item/, accessed on 26 November 2021), and researchers in Iran, the United Kingdom, and other regions where the spread of the disease was serious made urgent proposals for countermeasures [8,108]. As a result of the spread of COVID-19 infection, people refrained from going out, which reduced opportunities for exercise and training, and there were concerns about the decline in immune function as well as physical weakness [8,109,110].

Proper nutrition and exercise have no alternative to support immune function, whereas dietary restriction, such as fasting, is known to have positive effects, such as prolonging life span, improving insulin sensitivity, reducing OS and inflammation, and decreasing mortality from cancer and cardiovascular diseases. It is recently reported that, with regard to immune function, a 3-day fast has positive effects to improve it [108]. However, during the COVID-19 epidemic, both strenuous exercise and training is dangerous while fasting, because exercise under fasting conditions not only causes exhaustion and dehydration, but also leads to OS, inflammation, muscle damage, and fatigue, and increases the possibility of becoming infected. Therefore, the precautions for training during the month-long fasting period (Ramadan), a religious event for Muslims, including the intensity, duration, and frequency of exercise, as well as the recommended timing of food intake and nutrient requirements, were presented [108,110]. 

**Figure 3:**
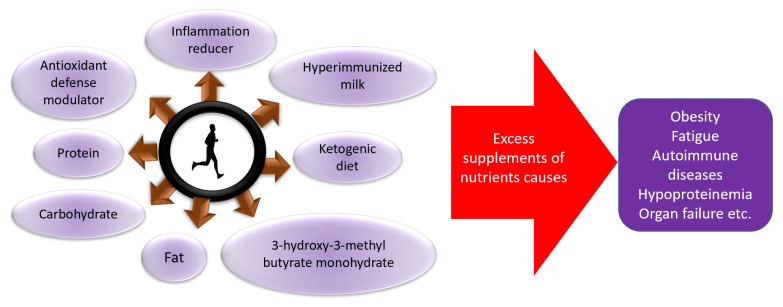
Role of nutrients for increased or decreased exercise performance and health.

In order to maintain the immune system, it is important to maintain the amount of exercise that does not cause excessive stress to the body, and to provide adequate nutritional intake and rest [8,108,111,112,113,114,115,116,117] (Figure 3), which were also explained by a digest version in an easy-to-understand manner [118]. Furthermore, it is recently suggested that moderate exercise and physical activity is effective, even for the recovery from COVID-19, suggesting the importance of exercise in the rehabilitation of the disease as well [119,120].

## 7. Conclusions

In this article, recent applications of functional foods to sports nutrition—and their interface with immunology and inflammation research—were updated. Although many of the findings presented in this article are nascent and require additional research, those results may represent a “paradigm shift” in the area of food functionality because they indicate previously under-recognized roles for supplements in areas such as energy metabolism, antioxidant pathway and inflammation regulation, immune function improvement, and anti-fatigue effects. Though the scope, application, and handling of these results in sports contexts still require caution, it is expected that future work will verify the efficacy and safety of functional foods such as these in the future. In addition, although vaccination against COVID-19 has become a commonplace in many countries around the world, robust treatment methods have not yet been established at present, and it is essential to continue health promotion and disease prevention focusing on exercise and nutrition to boost immune functions including vaccination effectiveness. It is important that we continue to share information and cooperate internationally in order to make progress in order to solve these problems.
